# Phylostratigraphic Analysis Reveals the Evolutionary Origins and Potential Role of New Genes in the Adaptive Evolution of *Spodoptera frugiperda*

**DOI:** 10.3390/ijms27010549

**Published:** 2026-01-05

**Authors:** Yi Yang, Bo Zhang, Yaobin Lu, Xinyang Zhang

**Affiliations:** 1 Xianghu Laboratory, Hangzhou 311231, China; 2 Institute of Insect Sciences, Zhejiang University, Hangzhou 310058, China

**Keywords:** *Spodoptera frugiperda*, new genes, adaptive evolution, phylostratigraphic analysis, co-expression network

## Abstract

The fall armyworm, *Spodoptera frugiperda*, has become one of the most damaging agricultural pests worldwide, yet the genetic basis of its extraordinary adaptability remains elusive. Recent studies have highlighted the pivotal role of newly evolved genes in adaptive evolution, and phylostratigraphy has emerged as a powerful conceptual framework to trace their origins. Here, we adopt this framework to investigate how new genes have contributed to the rapid adaptive evolution of *S. frugiperda*. Using high-quality genomic data, we inferred gene ages across evolutionary phylostrata and identified 277 newly evolved genes that originated after the divergence of *Spodoptera*. These new genes exhibit hallmark genomic signatures of recent origin, including shorter coding regions, simplified structures, and relaxed evolutionary constraints. Interestingly, transcriptomic analyses revealed strong tissue specificity, with pronounced enrichment in the antenna and brain, indicating possible involvement in chemosensory and neural functions essential for environmental and behavioral adaptation. Under diverse environmental challenges such as pesticide and parasitoid wasp exposure, and virus infection, we found many of the new genes acted as hubs in the regulatory networks associated with pesticide response. Together, our findings suggest that the emergence of new genes has played a critical role in shaping the rapid adaptive evolution of *S. frugiperda* and provide broader insights into how newly evolved genes contribute to species adaptation.

## 1. Introduction

The fall armyworm (FAW), *S. frugiperda*, is a highly destructive and migratory lepidopteran pest that has triggered global agricultural emergencies [1,2,3]. Since its transcontinental invasion from the Americas into Africa in 2016, followed by rapid spread across Asia [4,5], FAW has become a serious threat to global food security. It now affects more than 100 countries worldwide and causes devastating yield losses in major crops such as maize, rice, cotton, and sugarcane [6]. Classified by the Food and Agriculture Organization of the United Nations as a top-ten plant pest, FAW exhibits remarkable polyphagy, feeding on over 350 host plant species [7,8]. Its exceptional migratory capacity, aided by prevailing wind currents, and high reproductive potential enable rapid population outbreaks that cause substantial economic losses [9]. For example, estimation of the potential economic loss of maize in China caused by FAW indicates a range from US $5.4–47 billion per year [10]. The extraordinary ecological success of FAW across diverse agroecological systems underscores its profound adaptability and highlights an urgent need to elucidate the molecular mechanisms that drive its rapid adaptive evolution.

New genes, defined as those that originated relatively recently in evolutionary history, are increasingly recognized as key contributors to lineage -specific innovations and adaptive traits [11]. The rapid accumulation of high-quality genome assemblies across the tree of life now enables systematic inference of gene age and the discovery of lineage-specific new genes through comparative phylogenomic approaches [12,13,14]. Genome-wide studies in animals and plants have revealed that new genes can originate through mechanisms such as gene duplication [15], retroposition [16,17], and de novo emergence from noncoding DNA [18], and that many of these genes rapidly acquire essential biological functions [19]. For instance, the de novo gene *SCREP* in roses gradually evolved a complete protein-coding structure from noncoding sequences and now regulates volatile compound biosynthesis that shapes floral scent diversity [20]. In bamboo, 19 de novo genes are linked to rapid shoot growth [21], while in insects such as *Drosophila*, newly originated genes, including *COX4L* and *Zeus*, are indispensable for male fertility [22,23]. Similarly, in parasitoid wasps, a young gene integrated into the venom regulatory network demonstrates how new genes can be rapidly co-opted into critical physiological pathways [24]. Collectively, these studies illustrate that newly evolved genes are not evolutionary byproducts but can serve as drivers of adaptive phenotypic diversification [11,25,26]. However, whether such new genes contribute to the remarkable environmental adaptability and invasion success of *S. frugiperda* remains largely unknown.

In this study, leveraging a high-quality genome assembly of *S. frugiperda*, we systematically inferred gene ages through genomic phylostratigraphic analysis and identified 277 new genes that originated after the divergence of *Spodoptera*. Comparative analyses revealed that these new genes exhibit distinct genomic features compared with older genes, including shorter coding sequences, simplified exon–intron structures, and reduced evolutionary constraints. Transcriptomic profiling further demonstrated that new genes exhibit tissue-specific expression patterns. In addition to reproductive organs, many of these genes are preferentially expressed in the antenna and brain, suggesting potential roles in chemosensation, neural activity, and behavioral adaptation that may be crucial for host recognition and invasion success. Moreover, by integrating extensive RNA-seq datasets under various environmental conditions, including parasitoid wasp exposure, viral infection, host plant changes, and pesticide treatments, we found that new genes are frequently co-opted as regulatory hubs within modules associated with pesticide responses. Our findings provide a comprehensive catalogue of new genes in FAW and uncover their potential contributions to its extraordinary adaptability. This study represents the first phylostratigraphy-guided, systems-level analysis linking newly evolved genes to pesticide adaptation in an invasive insect. It provides a valuable genomic framework for investigating the molecular mechanisms of adaptive evolution and advances our understanding of how new genes contribute to rapid adaptation in pest species.

## 2. Results

### 2.1. Origin and Features of New Genes in S. frugiperda

**Figure 1:**
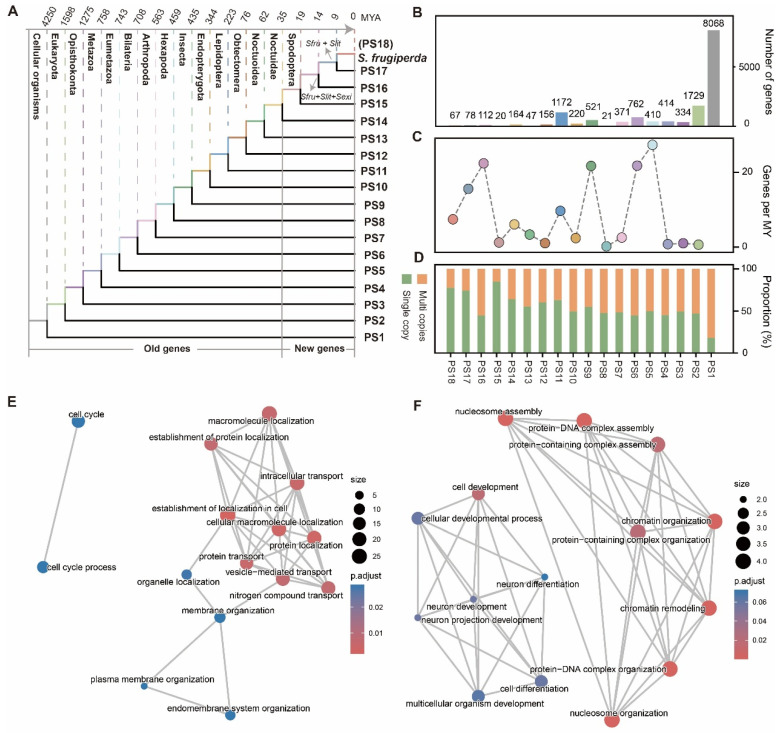
Phylostratigraphic analysis inferring gene ages in *S. frugiperda*. (**A**) Distribution of phylostratigraphic ages in *S. frugiperda*. Divergence times for each phylostratum were obtained from TimeTree [32]. Genes that originated after the Spodoptera lineage divergence (PS15–PS18, ~35 million years ago) were defined as “new genes.” (**B**–**D**) The number of genes (**B**), gene birth rate (**C**), and distribution of single-copy versus multicopy genes across phylostrata. (**E**,**F**) Gene Ontology (GO) enrichment analysis of old genes (PS2; (**E**)) and new genes (**F**).

To investigate the evolutionary origins of genes in *S. frugiperda*, we analyzed its chromosome-level reference genome assembly (NCBI RefSeq: GCF_023101765.2). This genome is of high quality and completeness, with a scaffold N50 of 13 Mb and a BUSCO completeness score of 99.3% (Table S1). We performed a genomic phylostratigraphy analysis to infer the evolutionary age of each gene [27,28]. Because phylostratigraphy relies on the NCBI Taxonomy database to establish a hierarchical framework [27], the immediate phylogenetic context of *S. frugiperda* is the genus *Spodoptera*. To improve the resolution for recently evolved, lineage-specific genes (i.e., those originating from the ancestor of *S. frugiperda* and *S. litura*, or from the *S. frugiperda*–*S. exigua* split), we incorporated two additional high-quality *Spodoptera* genomes (*S. litura* and *S. exigua*; Table S1) [29,30]. The final phylogenomic framework comprised 18 phylostrata (PS) ranks, ranging from the origin of cellular organisms (PS1, oldest) to *S. frugiperda* linage (PS18, youngest) (Figure 1A).

Using the 14,679 protein-coding genes in *S. frugiperda* genome as queries, we successfully assigned a total of 14,666 genes (99.9%) to 18 phylogenetic ranks (Table S2). More than half of the genes (55%, 8068/14,679) were assigned to PS1, reflecting their ancient origin in early cellular organisms before 4250 MYA (Figure 1B). This observation is consistent with patterns reported in many other lineages, including human, fruit fly and *Populus*, where a large fraction of genes trace back to ancient origins [14,31]. Gene birth numbers showed a strong positive correlation with evolutionary time (*R* = 0.94, *p* = 8.92 × 10^−9^), suggesting a continuous accumulation of genes throughout evolutionary history. We next calculated the gene birth rate at each time period, and found three peaks with gene birth rate significantly higher than the mean gene birth rate of 1.55 genes per MY, corresponding to PS5–PS6 (Eumetazoa–Bilateria, ~37.3 genes per MY), PS9–PS8 (Insecta–Endopopteryota, ~21.7 genes per MY) and PS16–PS15 (Spodoptera—the common ancestor of *S. frugiperda* and *S. litura*, ~22.4 genes per MY), respectively (Figure 1C). Furthermore, we detected a positive association (Spearman’s ρ = 0.72, *p* = 8.5 × 10^−4^) between gene age and copy number classification (single copy vs. multi copies). The proportion of multicopy genes gradually increased with evolutionary depth. Only ~22% of genes were multicopy at the youngest phylostrata (PS18, ~9 MYA), but this proportion rose through intermediate stages (~33–55%) and reached ~82% in the oldest phylostratum (PS1, ~4250 MYA) (Figure 1D). Thus, younger genes are more likely to be single copy. This pattern has also been reported in several other eukaryotic lineages, where lineage-specific genes are often orphan genes lacking detectable paralogs.

We defined genes that originated from PS15 as “new genes” (i.e., *Spodoptera*-specific genes, estimated age of 35 MYA). A total of 277 new genes were identified, including 67 genes that arose after the divergence between *S. frugiperda* and *S. lit* (i.e., *S. frugiperda*-specific genes, ~9 MYA). To explore the potential functions of these newly evolved genes, we performed functional annotations and found limited assignments (Figure S1), likely due to their young age and the absence of orthologs in current functional databases that rely on sequence homology. Gene Ontology (GO) enrichment analysis showed that, compared with older genes (e.g., those originating at PS2, Eukaryota, ~1598 MYA) that were significantly associated conserved cellular functions such as cell cycle, protein transport and plasma membrane orgnization, new genes were enriched for categories related to chromatin organization, cell development and neuron development (Figure 1E,F). Notably, despite these enrichments, the majority of new genes (95%, 264/277) lacked GO annotation (Figures S1 and S2). This suggests that while a subset may participate in specific biological processes, most remain functionally uncharacterized and could contribute to lineage-specific phenotypic innovations, warranting further investigation.

### 2.2. Fewer Exons and Weaker Selective Constraints in New Genes

**Figure 2:**
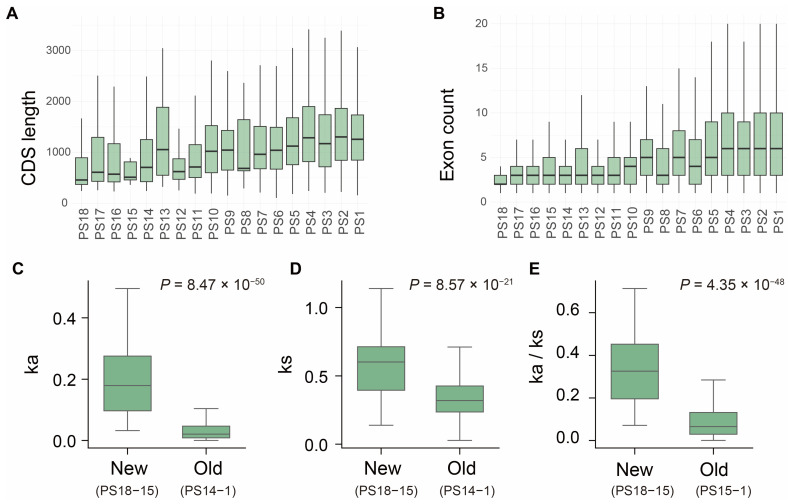
Structural characteristics and selection pressures of genes in *S. frugiperda* across evolutionary ages. Box plots showing the distributions of CDS length (**A**) and exon number (**B**). (**C**–**E**) Comparison of nonsynonymous substitution rate (Ka, (**C**)), synonymous substitution rate (Ks, (**D**)), and the ratio of nonsynonymous to synonymous substitution rates (Ka/Ks, (**E**)) between new genes (PS18–15) and old genes (PS14–1). *p* value was calculated using a one-sided Wilcoxon rank-sum test.

Typically, newly emerged genes will experience multiple evolutionary processes before reaching fixation since their birth, which may lead to distinctive characteristics compared to older genes, such as differences in gene length, exon-intron architecture, and evolutionary rate [18,21,33,34]. To explore whether these features of genes in *S. frugiperda* are associated with gene age, we examined 14,666 genes across the 18 phylostrata ranks. We first investigated the relationship between the coding sequence (CDS) length and gene age. Our results revealed that newly evolved genes generally possess shorter CDS (Figure 2A). The average CDS length of new genes (PS15–PS18) was 1042 bp, which is significantly shorter than the expected length of 1648 bp, based on a permutation test (*n* = 10,000, *p* < 0.05). This length was also shorter than that of the older genes originating from PS1 (average length = 1782; *p* = 6.3 × 10^−45^, one-sided Wilcoxon rank-sum test; Figure 2A). Moreover, we observed a positive correlation between CDS length and gene age (*R* = 0.616, *p* = 0.0085; Figure S3), suggesting that coding regions gradually expand as genes evolve over time.

We next examined the gene structural features of these new genes, hypothesizing that new genes tend to possess simpler architectures. We observed that the exon number progressively declined with decreasing gene age (Figure 2B). Specifically, the new genes dispersed in generally harbored fewer exons (average = 4.09) compared to the oldest genes in PS1 (average = 8.3, *p* = 1.4 × 10^−33^, one-sided Wilcoxon rank-sum test). The exon–intron architecture likely reflects the mechanism by which a gene originated. For example, genes derived from retro-transposition or de novo emergence often exhibit single- or few-exon structures, whereas those arising from DNA-based duplication may inherit exon–intron patterns from their parental genes [14]. Consistent with our previous observations, most newly evolved genes in *S. frugiperda* are single-copy (i.e., orphan) genes that lack paralogs, which may contribute to their reduced exon numbers. Indeed, 21 out of 277 newly emerged genes exhibited single-exon structures.

Beyond structural differences, the evolutionary trajectories of genes are also influenced by selective pressures [11,35,36]. Thus, we further assess the evolutionary constraint of the *S. frugiperda* genes across different phylostrata by calculating the Ka, Ks, and Ka/Ks ratio for 9741 orthologs shared with the closely related species *S. litura*. In contrast with the older genes ranging from PS1 to PS14, the new genes in PS15–18 displayed higher Ka, Ks, and Ka/Ks ratios with increasing phylostratum age (*p* = 8.47 × 10^−50^, 8.57 × 10^−21^, and 4.35 × 10^−48^, respectively; one-sided Wilcoxon rank-sum test; Figure 2C–E). This suggests that new genes have undergone fewer evolutionary constraints, whereas those in older genes have evolved under stronger purifying selection.

In summary, our results indicate that gene evolution in *S. frugiperda* follows a gradual trajectory in which new genes possess shorter coding regions, simpler exon–intron structures, and weaker selective constraints, while these features become progressively elaborated with increasing evolutionary age.

### 2.3. New Genes Exhibit Tissue-Specific Expression Patterns

The fate of newly emerged genes is thought to involve rapid changes in their sequence, structure, and expression profiles to avoid degeneration into pseudogenes. Functional expression is often regarded as critical evidence of gene fixation, implying ongoing adaptive evolution [37,38,39]. To investigate the potential functional divergence and expression characteristics of new genes in *S. frugiperda*, we analyzed 38 transcriptome datasets derived from various tissues, including the brain, antenna, thorax, ovary, and pheromone gland of adult *S. frugiperda*, as well as prothoracic gland (PG), hemocytes, salivary glands, male gonads, cuticle, midgut, Malpighian tubules, and body wall cells (BCCs) from larvae (Table S3). In addition, 27 transcriptomes from different developmental stages, ranging from 1st to 6th instar larvae, pupae, and adults, were included (Table S4).

**Figure 3:**
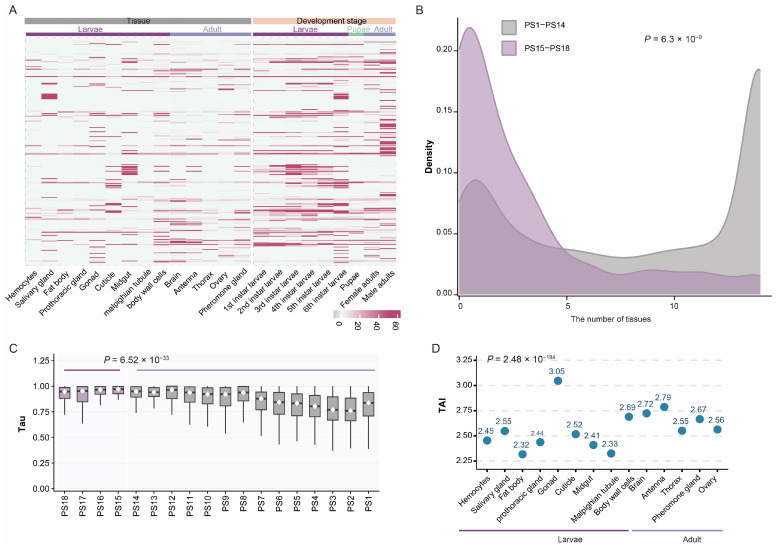
Expression patterns of new genes in *S. frugiperda*. (**A**) Heatmap showing the expression patterns of new genes across various tissues and developmental stages. (**B**) Distribution of expressed tissues for new genes (purple) and old genes (gray). (**C**) Distribution of Tau scores across the 18 phylogenetic ranks. (**D**) Transcriptome age index for each tissue of *S. frugiperda*, suggesting that new genes are preferentially enriched in the gonad of male larvae, and in the antenna and brain of adult *S. frugiperda*.

We first quantified the expression levels of all 14,666 annotated genes in the *S. frugiperda* genome. The vast majority (95%, 13,948/14,666) showed detectable expression (TPM > 1) in at least one sample. Notably, 90% (250/277) of the new genes we identified were expressed in at least one dataset (Figure 3A). Although the proportion of expressed new genes was not significantly higher than expected (*p* = 0.999, hypergeometric test), the observation that most new genes are transcriptionally active suggests that they may have functional roles. Moreover, both the proportion of expressed genes and their expression intensity increased with evolutionary age, indicating that older genes are more likely to be broadly and highly expressed, likely reflecting their fundamental biological functions.

We next investigated whether new genes are broadly or specifically expressed across tissues. To assess this, we quantified each gene’s expression breadth by counting the number of tissues in which it was expressed. Roughly half of all genes (7022/14,666) were broadly expressed (i.e., expressed in ≥10 tissues). However, compared with older genes (average tissues expressed = 8.08), new genes in *S. frugiperda* tended to exhibit more restricted, tissue-specific expression patterns (average tissues of the new genes expressed = 2.81; *p* = 6.3 × 10^−45^, one-sided Wilcoxon rank-sum test; Figure 3B). Because simple tissue counts may be biased by expression magnitude (e.g., genes highly expressed in one tissue but weakly in others), we further calculated the tissue-specificity index (Tau). Consistent with the previous result, new genes displayed significantly higher Tau values than older genes (average = 0.879, *p* = 6.52 × 10^−33^, one-sided Wilcoxon rank-sum test; Figure 3C), confirming their generally stronger tissue specificity. Examination of tissue-specific genes (tau > 0.8) revealed that new genes are particularly enriched in the brain and midgut (*p* = 6.3 × 10^−9^, chi-square test).

To further integrate gene age and expression profiles, we computed the transcriptome age index (TAI) for each *S. frugiperda* tissue. The male larval gonad exhibited the highest TAI (3.05, Figure 3D), indicating a strong enrichment of young gene expression. This pattern is consistent with findings in *Drosophila*, where the testis is recognized as a major “birthplace” of new genes [40]. In addition to male gonads, surprisingly high TAI values were also observed in the antenna (2.79) and brain (2.72), further supporting the notion that new genes tend to be expressed in a tissue-specific manner.

### 2.4. New Genes Contribute to Adaptive Responses to Pesticide Exposure

**Figure 4:**
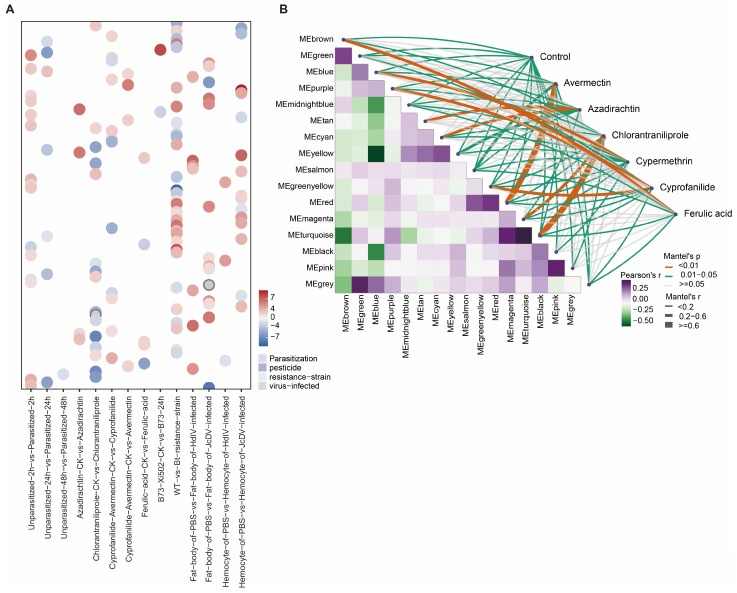
New genes contribute to the adaptive evolution of *S. frugiperda*. (**A**) Distribution of log_2_FC of differentially expressed new genes in each treatment group relative to the control. Positive and negative values indicate upregulation and downregulation, respectively. (**B**) Gene co-expression networks constructed under different pesticide treatments.

The observation that new genes are functionally expressed and often exhibit tissue-specific expression prompted us to examine whether they participate in the adaptive evolution of *S. frugiperda*. To further explore this possibility under ecologically relevant conditions, we analyzed 111 RNA-seq datasets obtained from four types of environmental challenges, including parasitization, pesticide exposure, virus infection and feeding on diverse insect-resistant host plants (Table S5). Across 21 experimental comparisons, we identified 9322 differentially expressed genes (DEGs). Among these, 136 new genes exhibited differential expression in 20 experimental contrasts involving parasitization, pesticide exposure, viral infection, and feeding on diverse host plants compared to their respective controls (Figure 4A and Figure S4).

The largest number of new gene DEGs was observed in response to pesticide exposure. Specifically, 51 of the 136 new genes showed significant expression changes in the comparison between Chlorantraniliprole-treated and control samples. In other pesticide treatments, 40, 35, and 31 new genes were differentially expressed following exposure to Azadirachtin, Cyprofanilide, and Avermectin, respectively, with more than half of them being upregulated. A similar pattern was observed under viral infection, where over 90% of the new genes were upregulated in individuals infected with JcDV or HdIV relative to uninfected controls. Despite their recent evolutionary origin, these findings indicate that new genes are transcriptionally responsive to environmental stressors, particularly chemical insecticides, implicating them in adaptive processes.

Following their emergence, new genes can be co-opted into conserved regulatory modules, where they interact with pre-existing genes to perform specialized functions. To further elucidate the potential biological roles of newly evolved genes in pesticide responses, we constructed a gene co-expression network using transcriptomic data from *S. frugiperda* exposed to Avermectin, Azadirachtin, Chlorantraniliprole, Cypermethrin, Cyprofanilide, and ferulic acid. Among the five highly correlated co-expression modules identified (MEred, MEtan, MEgreenyellow, MEpurple, and MEbrown; kME > 0.6), the MEturquoise module showed the strongest association with the Chlorantraniliprole response (*R*^2^ = 0.89, *p* < 0.05). Within this module, 79 differentially expressed new genes were co-expressed with 5339 older differentially expressed genes (DEGs) (PS1–PS14) (Figure 4B). Notably, 26 of these new genes exhibited kME values greater than 0.7, suggesting that they may function as potential hub genes together with older genes in the co-expression network (Figure S5).

Together, these results demonstrate that new genes are integrated into cooperative gene networks and actively contribute to environmental adaptation, particularly pesticide resistance, in *S. frugiperda*. Their involvement highlights the evolutionary significance of gene innovation as a driver of rapid adaptive responses.

## 3. Discussion

As biological organisms evolve, the gene repertoire within their genomes are far from constant, with frequency gain and loss events shaping genomic diversity [41,42,43]. Each gene has its own origin and evolutionary age, and understanding when and how genes emerged is essential to uncovering the driving forces of species adaptation [11,44]. Recent advances in sequencing technology have provided unprecedented opportunities for systematically inferring the evolutionary age of genes at a high resolution [11]. Here, using the high-quality genome of *S. frugiperda* [45,46], we successfully inferred the age groups of 14,666 genes through phylogenomic analysis. Among them, we identified 277 newly evolved genes that originated after the divergence of *Spodoptera*. The distribution of gene ages in *S. frugiperda* is similar to that observed in other lineages, as in plants and mammals [14,21,31], with roughly half of the genes tracing back to ancient origins, approximately 4250 million years ago, predating the earliest cellular organisms. This pattern reflects a general evolutionary trajectory shared across life, from prokaryotes to hominids, where increasing biological complexity is mirrored by the continuous diversification of genomic repertoires.

A long-standing question in evolutionary biology is whether ancient, conserved genes are more functionally important than newly evolved ones [11]. Recent studies have highlighted the essentiality of new genes, showing that they can rapidly acquire indispensable roles in species survival and adaptation [22,47,48]. For example, the discovery of *katherine johnson* (*kj*) reveals a novel aspect of male fertility controlled by a gene that acts during spermatogenesis but whose functional consequence is manifested in the efficient entry of sperm into the egg [49]. Our results revealed a similar pattern in *S. frugiperda*. Older genes tend to participate in fundamental biological processes, particularly related to the fundmental cellular functions, whereas newly evolved genes are more likely to be associated with specific functions (Figure 1E,F). However, it is worth noting that the majority of the newly evolved genes in *S. frugiperda* remain functionally uncharacterized, making them promising targets for future investigation. Given the worldwide impact of *S. frugiperda* as a pest species [6,49,50], these new gene datasets offer valuable resources for exploring the molecular basis of adaptation.

The expression patterns of new genes in *S. frugiperda* further support their potential roles in adaptation. Generally, new genes exhibit restricted expression, being highly expressed in one or a few tissues compared with older genes (Figure 3B). This pattern aligns with observations across diverse taxa, including *Drosophila*, humans, as well as plants, including bamboo and rose [20,21,51]. Previous studies have shown that many new genes show testis-biased expression, consistent with the “out of the testis” hypothesis, which posits that the testis serves as a cradle for new gene emergence before these genes gradually acquire broader functional roles [40,52,53,54]. In *S. frugiperda*, we also observed significant enrichment of new gene expression in the gonads of male larvae. Unexpectedly, we also detected high transcriptome age index (TAI) values in the antenna and brain, suggesting that new genes are also enriched in these two tissues (Figure 3D). Consistently, an independent analysis of tissue specificity revealed a similar pattern, with tissue-specific new genes being preferentially expressed in the brain. This observation aligns with our functional analysis, which showed that new genes are enriched for neuronal functions (Figure 1F). Together, these findings suggest that new genes may play important roles in sensory perception and neural processes. Given that the antenna and brain are key organs for environmental sensing and behavioral regulation in insects, these findings imply that new genes may contribute to the adaptive evolution of *S. frugiperda* through environmental and behavioural responsiveness.

As *S. frugiperda* is a globally notorious pest due to its inherent ability to survive in a wide range of habitats, its strong migration ability, high fecundity, rapid development of resistance to insecticides/viruses and its gluttonous characteristics [55], understanding the genetic mechanisms underlying its adaptability is of great importance. New genes have been shown to play crucial roles in adaptive evolution, either directly [56,57] or through participation in gene regulatory networks [21,24,58,59]. Using RNA-seq data from *S. frugiperda* exposed to four treatments including parasitization, pesticide exposure, viral infection, and feeding on diverse host plants, we found that new genes exhibited widespread differential expression across most stress conditions, except noru viral infection. The strongest differential expression was observed under pesticide exposure. Further weighted gene co-expression network analysis (WGCNA) revealed that a substantial proportion of new genes were incorporated into modules associated with pesticide metabolism and detoxification, with several acting as hub genes. This suggests that new genes have evolved to participate in the molecular pathways underlying pesticide resistance, potentially contributing to the remarkable environmental adaptability of *S. frugiperda*.

Several caveats of our study should also be noted. First, our gene age inference pipeline relies on the completeness of available genomic databases and the quality of current gene annotations. As more high-quality genomes become available and whole-genome alignment–based approaches continue to improve, the accuracy and resolution of gene age estimation are expected to increase. Second, although we conducted a thorough functional annotation of the newly identified genes, the majority remain uncharacterized. Nevertheless, their distinct expression patterns across specific tissues and stress conditions provide a valuable framework for prioritizing candidates for future functional and mechanistic studies. Third, our observation of several new genes occupying central positions within pesticide-responsive co-expression modules likely reflects regulatory innovations, driven by the integration of new genes into existing gene networks. However, it remains unclear at which level these new genes act and warrants further exploration through comparative network analyses or experimental validation.

In summary, we inferred the evolutionary ages of 14,666 genes in the *S. frugiperda* genome and revealed distinct structural and expression features of newly evolved genes. Our findings highlight the potential roles of these genes in adaptive evolution, particularly in pesticide resistance, and provide an important genomic resource for future research on the molecular basis of adaptation in *S. frugiperda*. From an applied perspective, insights into how new genes facilitate rapid stress adaptation could inspire new strategies in pest management and even broader biomedical contexts, such as anti-tumor biology. More broadly, this work offers insights into the evolutionary dynamics of new genes and their contributions to species diversification and environmental adaptation.

## 4. Materials and Methods

### 4.1. Genomic and Transcriptomic Data

The high-quality genome data of *S. frugiperda* (GCF_023101765.2), *S. litura* (GCF_002706865.2), and *S. exigua* (GCA_902829305.4) were collected from the National Center for Biotechnology Information (NCBI). Transcriptomic data from 38 tissue samples of *S. frugiperda*, including hemocytes, salivary gland, fat body, prothoracic gland, gonad, cuticle, midgut, malpighian tubule and body wall cells of larvae, as well as antenna, thorax, pheromone gland, ovary and brain of adults, were downloaded from NCBI SRA datasets. Additionally, 27 RNA-seq datasets covering larval-to-adult developmental stages and 111 RNA-seq samples from experiments involving parasitization, chemical pesticides, virus infection and feeding on diverse host plants were collected. All of these data resources were detailed in Table S6.

### 4.2. Phylostratigraphic Analysis

The longest isoform for each gene in the *S. frugiperda* genome was selected for downstream analysis using custom Python (v3.10) scripts. The Non-Redundant (NR) Protein Sequence Database and corresponding taxonomy datasets (retrieved date: September 2025) were retrieved from the NCBI. We used the GENERA v1.4.2 [41] pipeline to infer the gene age of each gene in *S. frugiperda*. This framework significantly enhances traditional phylostratigraphy by integrating gene family clustering and homology detection failure (HDF) correction, enabling robust identification of gene-family founder events while controlling for systematic biases such as sequence divergence and database incompleteness. Briefly, the pipeline operates through four key steps: (1) Homology search and taxonomic mapping: all protein sequences of the query species were aligned against the NR protein database using DIAMOND v2.14 [60] in sensitive mode. The resulting sequence matches were then mapped to their corresponding hierarchical lineages in the NCBI taxonomy database to establish evolutionary relationships. (2) Taxonomic trace-back and representativeness filtering: the most distant homolog for each gene was identified. A taxonomic representativeness score was calculated; genes with a score below 30% were flagged as potential horizontal gene transfers or contaminants and excluded from subsequent analysis. (3) Gene family clustering: paralogous genes were clustered into families using the Markov Cluster Algorithm (MCL) [61] based on an all-vs-all DIAMOND search of the proteins. The oldest reliable age among cluster members was assigned as the family’s founder age. (4) HDF probability estimation: For each gene, the probability that its non-detection in deeper taxa was due to HDF was computed using abSENSE [62]. Gene ages were considered high-confidence founder events if the HDF probability in the closest outgroup fell below 0.05.

For each phylostratum, genomic features, including exon number, CDS length, and gene length, were calculated using in-house Python scripts. Putative orthologs between *S. frugiperda* and *S. litura* were determined using reciprocal best hits by BLASTP v2.16.0 [63]. To assess the selection pressures acting on protein-coding sequences, the nonsynonymous (Ka) and synonymous (Ks) substitution rates, as well as the Ka/Ks ratio for 10,361 orthologous gene pairs, were subsequently computed using ParaAT v2.0 [64] and KAKS_Calculator v2.0 [65] under the YN00 model.

### 4.3. Transcriptomic Data Processing

Trim_Galore v0.6.10 (https://github.com/FelixKrueger/TrimGalore, accessed on 20 September 2025) was used to process the raw RNA-seq data to remove low-quality reads and adapter sequences with the parameters “--fastqc --quality 20 --length 20”. We then quantify the expression level of each gene based on the cleaned reads using Salmon v1.10.3 [66] under the “--validateMappings” mode, employing a gtf file generated from the original genome annotation via gffread v0.11.6 (https://github.com/gpertea/gffread, accessed on 20 September 2025).

Transcript abundance was quantified at both the isoform and gene levels, and normalized using the Transcripts Per Kilobase Million (TPM) metric [67]. According to the generated expression matrix for the 14 tissues, the tissue specificity of each gene was evaluated by calculating the tau score, implemented with the R (v4.5.0) package tispec (https://rdrr.io/github/roonysgalbi/tispec, accessed on 20 September 2025). Genes exhibiting a tau score greater than 0.8 were considered tissue-specific.

### 4.4. Transcriptome Age Index

The transcriptome age index (TAI) for each sample was computed using the myTAI v2.0.0 R package [68]. The TAI is defined as the weighted mean of the phylostratum ranks of all genes, with their respective expression levels serving as the weights. It is calculated using the formula:

$$TAI=\sum \left(PS\times e_{i}\right)/\sum e_{i}$$

where PS_i_ denotes the phylostratum of gene i, and e_i_ represents its expression value. A higher TAI value indicates that the transcriptome is enriched with evolutionarily younger genes, while a lower TAI value suggests a predominance of evolutionarily ancient genes [68].

### 4.5. Differential Gene Expression

Differential gene expression analysis across various treatments (parasitization, chemical pesticides, virus infection and feeding on diverse host plants) versus the control group was performed using the DESeq2 v1.46.0 package (https://github.com/thelovelab/DESeq2, accessed on 20 September 2025). Genes were considered significantly differentially expressed based on a threshold of a false discovery rate (FDR) adjusted *p* value of less than 0.05 and an absolute log2 fold change (|log2FC|) of at least 1.

### 4.6. Weight Gene Co-Expression Network

A weighted gene co-expression network was constructed from the gene expression profiles of 111 RNA-seq samples from the adaptive experiments using the WGCNA R package [69] with the parameters softPower = 8 and minModuleSize = 30. Prior to analysis, genes with zero expression (TPM = 0) across all samples were filtered out from the raw expression matrix. The treatment and control conditions were incorporated as phenotypic traits. To identify biologically relevant modules, module-trait associations were assessed by calculating the Pearson correlation between each module eigengene (ME) and the traits. For genes within each module, the module membership (kME) was defined as the Pearson correlation between the gene’s expression profile and ME. Genes with high kME values were considered potential hub genes. Finally, the co-expression network was visualized using Cytoscape v3.9.1 [70].

### 4.7. GO Enrichment

Gene Ontology (GO) enrichment analysis was performed using the R package clusterProfiler v4.14.6 [71].

## Data Availability

The original contributions presented in this study are included in the article/Supplementary Material. Further inquiries can be directed to the corresponding author.

## Supplementary Materials

The following supporting information can be downloaded at: https://www.mdpi.com/article/10.3390/ijms27010549/s1.
