# Supplementary material for: Phylostratigraphic Analysis Reveals the Evolutionary Origins and Potential Role of New Genes in the Adaptive Evolution of Spodoptera frugiperda
Source: Int J Mol Sci. 2026 Jan 5;27(1):549. doi: 10.3390/ijms27010549 (PMC12786933; doi:10.3390/ijms27010549)
Supplement: Supplementary file 1 [file ijms-27-00549-s001.zip › supplementary_figures.pdf]

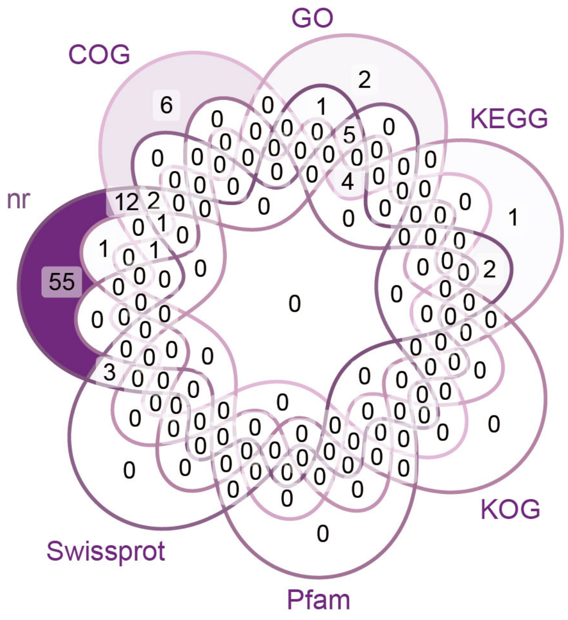

Figure S1. Functional annotation of the new genes

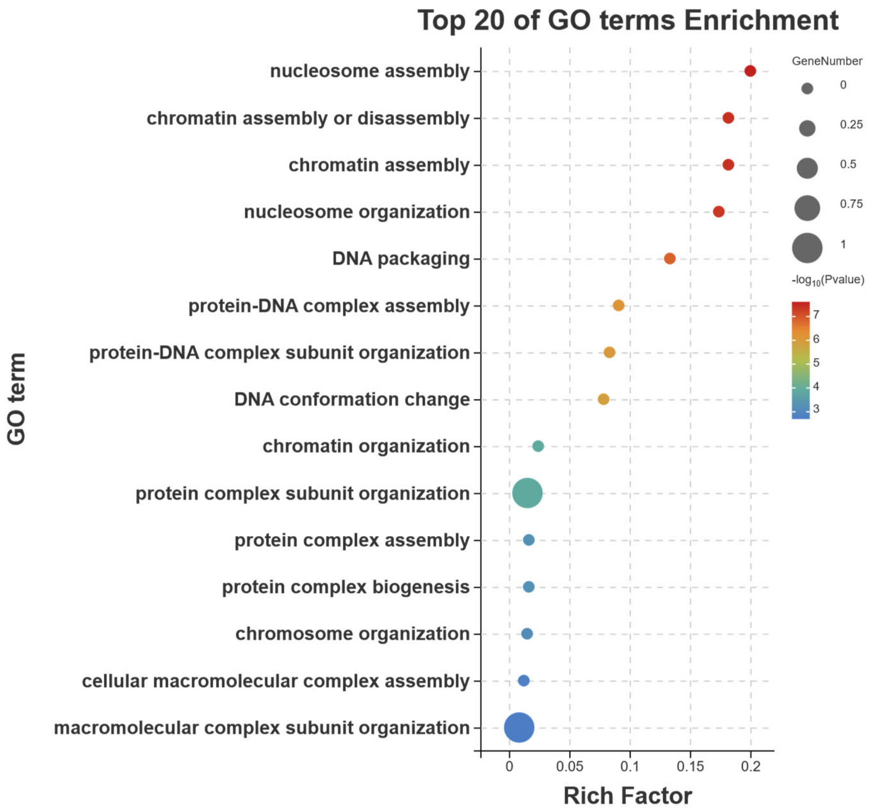

Figure S2. GO enrichment of the new genes

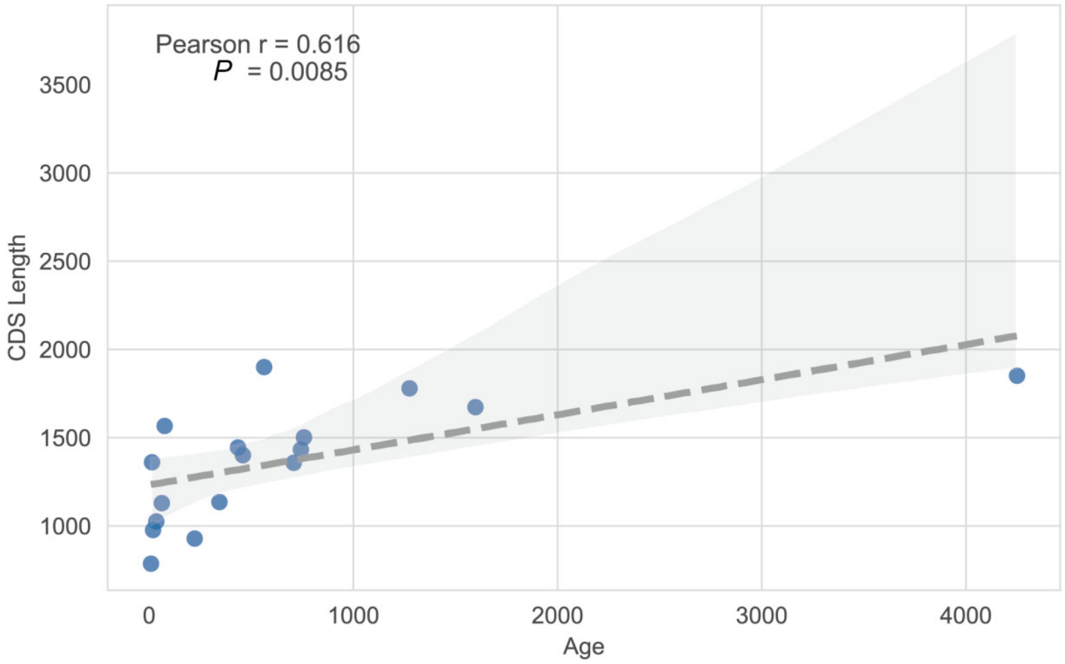

Figure S3. Correlation between CDS length and gene age

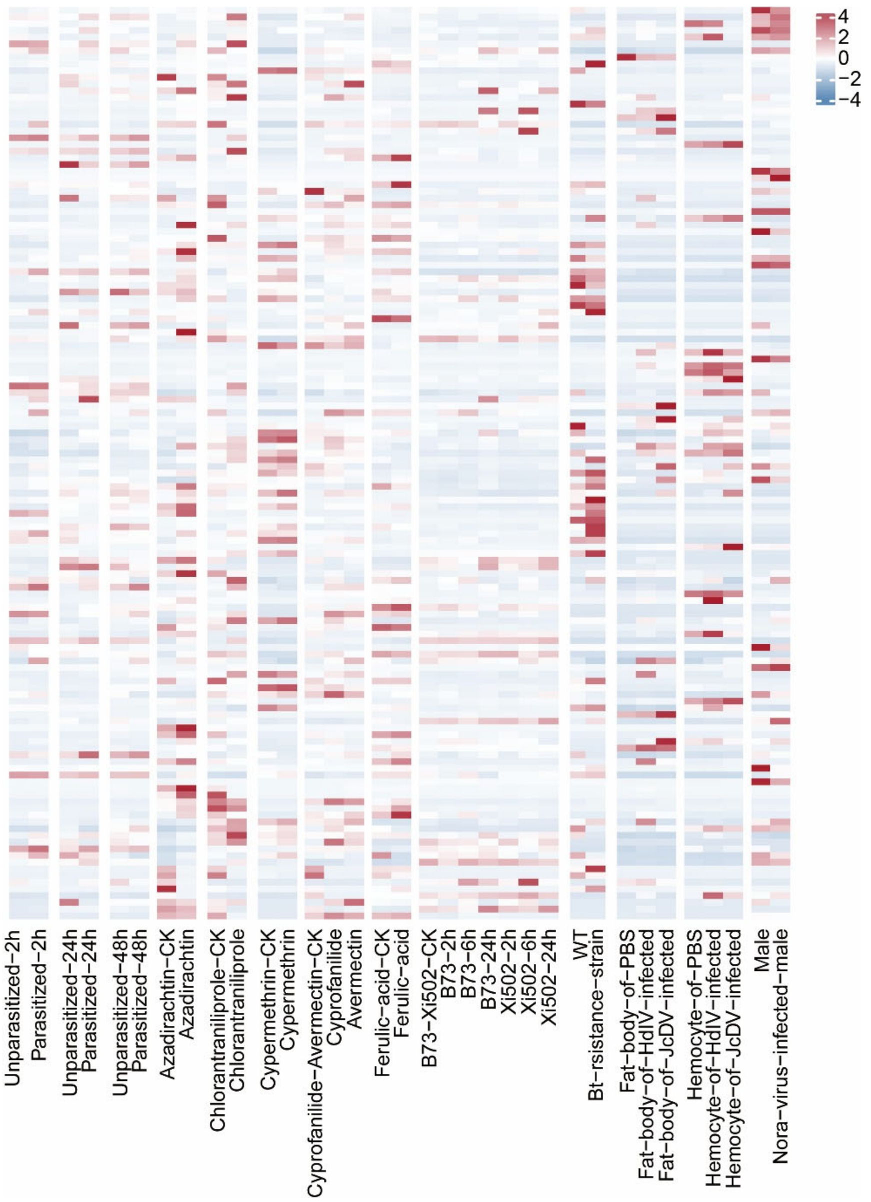

Figure S4. Expression levels of new genes in different treatment groups, including parasitization (purple), chemical pesticide exposure (dark purple), viral infection (light blue), and feeding on diverse host plants (gray), compared with the control group. Expression levels are shown as mean TPM values.

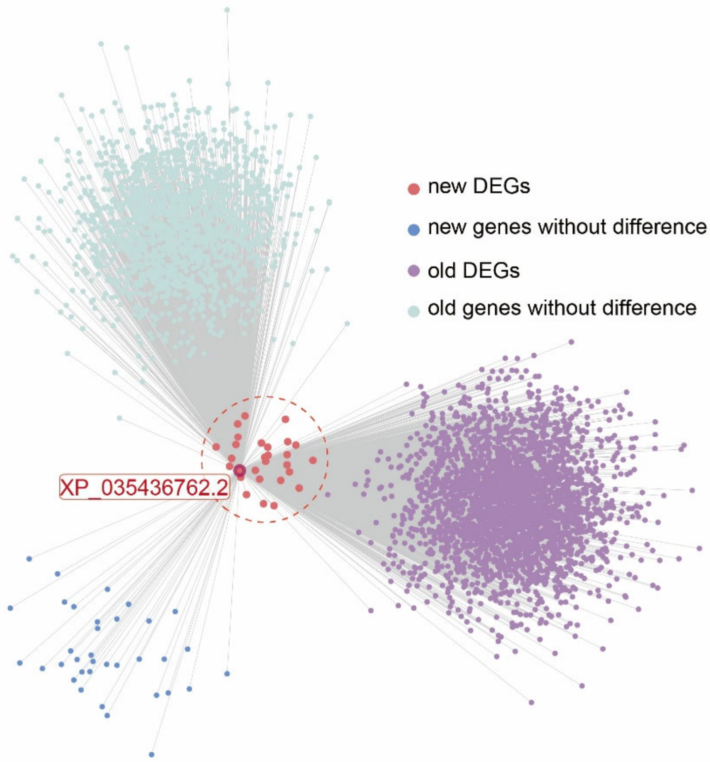

Figure S5. Detailed view of the gene network associated with the new gene XP_035436762.2. Node colors indicate gene categories: red, differentially expressed new genes; blue, non-differentially expressed new genes; purple, differentially expressed old genes; and green, non-differentially expressed old genes.
